# Application of ChatGPT4.o to Geriatrics 5Ms and Its Evaluation by Clinical Providers and Trainees

**DOI:** 10.1093/geroni/igaf122.1183

**Published:** 2025-12-31

**Authors:** Huai Cheng

**Affiliations:** University of Minnesota, Minneapolis, Minnesota, United States

## Abstract

ChatGPT did well on clinical vignettes from different disciplines. Our previous study showed ChatGPT had good geriatric knowledge and could help reduce polypharmacy and falls in two geriatric vignettes. Geriatrics 5M is a new geriatric framework and foundation for Age-Friendly Health System and the CMS new quality metrics for hospitalized older adults. This study aims to explore the responses to 8 geriatric 5Ms-based clinical questions by ChatGPT, clinical providers, and trainees. These 8 case-based clinical questions were integrated with two seminars on ChatGPT application to geriatric practice for 307 clinical providers and trainees. ChatGPT performance on these 8 geriatric 5Ms-based questions were evaluated by clinical providers and trainees. 8 geriatric 5Ms-based clinical questions were provided consequentially such as what is the simple instrument you will recommend evaluating the memory decline for this patient? What is the simple assessment instrument you will recommend assessing physical function for this patient? What is the simple instrument you will recommend evaluating potentially inappropriate medications (PIM) such as drug-drug, drug-disease interaction, and renal function adjusted medications? What is the simple instrument you will recommend predicting life expectancy for this patient? During 2 geriatric seminars, ChatGPT responded to these 8 questions well and were like clinical providers and trainees. More than 90% clinical providers’ and trainees’ responders agreed or strongly agreed with ChatGPT performance on 8 geriatrics 5Ms-based clinical questions. These results suggest ChatGPT could be applied to geriatric 5Ms practice as an assistant tool.

